# Digital counting of tissue cells for molecular analysis: the QuANTUM pipeline

**DOI:** 10.1007/s00428-024-03794-9

**Published:** 2024-03-26

**Authors:** Vincenzo L’Imperio, Giorgio Cazzaniga, Mauro Mannino, Davide Seminati, Francesco Mascadri, Joranda Ceku, Gabriele Casati, Francesca Bono, Catarina Eloy, Elena Guerini Rocco, Chiara Frascarelli, Matteo Fassan, Umberto Malapelle, Fabio Pagni

**Affiliations:** 1 Department of Medicine and Surgery, Pathology, IRCCS Fondazione San Gerardo Dei Tintori, University of Milano-Bicocca, Milan, Italy; 2 Pathology Laboratory, Institute of Molecular Pathology and Immunology of University of Porto (IPATIMUP), Porto, Portugal; 3 Pathology Department, Medical Faculty of University of Porto, Porto, Portugal; 4 Division of Pathology, European Institute of Oncology IRCCS, Milan, Italy; 5 Department of Oncology and Hemato-Oncology, University of Milan, Milan, Italy; 6 Surgical Pathology and Cytopathology Unit, Department of Medicine, DIMED, University of Padua, Padua, Italy; 7 Veneto Institute of Oncology, IOV-IRCCS, Padua, Italy; 8 Department of Public Health, University of Naples Federico II, Naples, Italy

**Keywords:** Molecular pathology, Computational pathology, Tumor cellular fraction, NGS, Non-small cell lung cancer

## Abstract

The estimation of tumor cellular fraction (TCF) is a crucial step in predictive molecular pathology, representing an entry adequacy criterion also in the next-generation sequencing (NGS) era. However, heterogeneity of quantification practices and inter-pathologist variability hamper the robustness of its evaluation, stressing the need for more reliable results. Here, 121 routine histological samples from non-small cell lung cancer (NSCLC) cases with complete NGS profiling were used to evaluate TCF interobserver variability among three different pathologists (pTCF), developing a computational tool (cTCF) and assessing its reliability vs ground truth (GT) tumor cellularity and potential impact on the final molecular results. Inter-pathologist reproducibility was fair to good, with overall W*k* ranging between 0.46 and 0.83 (avg. 0.59). The obtained cTCF was comparable to the GT (*p* = 0.129, 0.502, and 0.130 for surgical, biopsies, and cell block, respectively) and demonstrated good reliability if elaborated by different pathologists (W*k* = 0.9). Overall cTCF was lower as compared to pTCF (30 ± 10 vs 52 ± 19, *p* < 0.001), with more cases < 20% (17, 14%, *p* = 0.690), but none containing < 100 cells for the algorithm. Similarities were noted between tumor area estimation and pTCF (36 ± 29, *p* < 0.001), partly explaining variability in the human assessment of tumor cellularity. Finally, the cTCF allowed a reduction of the copy number variations (CNVs) called (27 vs 29, − 6.9%) with an increase of effective CNVs detection (13 vs 7, + 85.7%), some with potential clinical impact previously undetected with pTCF. An automated computational pipeline (Qupath Analysis of Nuclei from Tumor to Uniform Molecular tests, QuANTUM) has been created and is freely available as a QuPath extension. The computational method used in this study has the potential to improve efficacy and reliability of TCF estimation in NSCLC, with demonstrated impact on the final molecular results.

**Supplementary Information:**

The online version contains supplementary material available at 10.1007/s00428-024-03794-9.

## Introduction

In the precision medicine era, the assessment of molecular signatures of different cancer types is becoming of paramount importance for prognostic and predictive purposes [1, 2]. In this direction, emphasis goes to the dramatic change in the management of non-small cell lung cancer (NSCLC) in the last decade. The introduction of different targeted therapeutic options for NSCLC based on the detection of specific molecular alterations by next-generation sequencing (NGS) technologies increased the number of cases to be evaluated [3, 4]. Moreover, the progressive adoption of digital pathology in the diagnostic workflow [5–9] and of artificial intelligence (AI) tools for the simplification of repetitive, time-consuming, and poorly reproducible pathology tasks [10, 11] is guiding our discipline towards an holistic and integrative approach [12–14], with promising preliminary results even on the molecular characterization of NSCLC [15, 16]. One of the routine steps that can significantly benefit from computational algorithms is the evaluation of tumor cellular fraction (TCF), pivotal for adequacy assessment for molecular studies [17–19], and traditionally affected by significant inter-observer variability [20–23]. However, whether this computational evaluation may impact the final molecular analysis is still unknown and its applicability on more troublesome cancer specimens (e.g., cytology) has not been investigated yet. Thus, in the present study, we aim at applying a QuPath-based tool for the assessment of TCF on NSCLC cyto/histological specimens, evaluating the impact of this digital pathology approach on the final pathologist evaluation and the corresponding molecular results.

## Methods

### Cases

We prospectively enrolled NSCLC cases that underwent molecular analysis at the Oncological Molecular Pathology Unit of Fondazione IRCCS San Gerardo dei Tintori, Università degli studi di Milano-Bicocca (UNIMIB) in Monza, Italy, from October 2022 to August 2023. For each case, either histological or cell blocks (formalin-fixed and paraffin-embedded, FFPE) representative glass slides stained with hematoxylin and eosin (H&E) were selected by the molecular pathologist (path #1, DS) for the definition of tumor-containing regions (TCRs) that have been reported on the slide with pen-marks for subsequent tumor microdissection. At this time point, number of vital tumor cells (< or ≥ 100) and TCF (%) have been evaluated by the same pathologist (path #1, pTCF) and added to the final molecular pathology report for adequacy purposes. In particular, a TCF ≥ 20% and a value of viable tumor cells ≥ 100 were considered as the minimum desiderable prerequisites for the reliability of the subsequent molecular analysis [18, 24]. Slides were then blindly reviewed by a different pathologist (path #2, VL) and a pathology trainee (MM) for the assessment of pTCF. Obtained values were reported along with the original assessments for comparison purposes. The study has been approved by the local Ethics Committee (prot. 35859, 24/10/2022).

### Computational pathology pipeline

The retrieved slides scored by path #1 were anonymized and scanned with NanoZoomer S60 (Hamamatsu, Shizuoka, Japan) at 20 × magnification (0.4416 MPP) [6, 9]. The obtained WSI were imported in QuPath v0.4.4 [25], and subsequent analyses were restricted to the pen-marking area representative of the microdissected region (ROI). For each ROI, a cell-by-cell visual count and classification into “Tumor” or “Non-neoplastic” categories (e.g., immune cells, stromal cells, and normal epithelial cells) was obtained by two expert lung pathologists (FB and FP) and considered like the reference/ground truth (GT), as previously suggested (Supplementary Fig. 1) [23]. Subsequently, to obtain the computational TCF (cTCF), StarDist extension was used for nuclear detection (cells surrogate) as the main variable for the final cTCF evaluation [26], as described in the Supplementary Methods. Then, the QuPath machine learning (ML)–based algorithm (object classifier, Random Tree (RT), Supplementary Fig. 2) for nuclear assignment to “Tumor” vs “Non-neoplastic” was trained using manual annotations. These annotations included a balanced ratio of five cells for each category and reached the best performances at a maximum cutoff of 10 annotations (Supplementary Fig. 3). Additionally, an “Ignore” class was created for those detections that did not correspond to nuclei (e.g., artifacts, red blood cells, mucus, anthracosis). The obtained cTCF was used then for comparison with the human-obtained pTCF. To further investigate the possible differences between cTCF and pTCF, a deep learning (DL) approach (WSInfer QuPath extension [27]) has been used to automatically outline tumor foci boundaries (TF) from surrounding stroma (“Non-neoplastic/Other,” as detailed in Supplementary Methods). Finally, the reproducibility of the obtained cTCF was assessed on a subset of five randomly selected cases for each sample category (surgical, biopsy, cell block, *n* = 15), on which other two pathologists (CE and EGR, validator #1 and #2) were used as independent annotators, to evaluate the robustness and reliability of the proposed tool if used by different pathologists.

### Copy number variations (CNVs) and TCF

NGS occurred on the Ion Torrent™ Genexus™ System platform (Thermo Fisher Scientific), with the Oncomine™ Precision Assay (OPA, Thermo Fisher Scientific), capable of detecting 50 clinically relevant genes in NSCLC (Supplementary Methods). The computational analysis of sequencing data was performed before and after the cTCF scoring, to estimate possible changes in the final molecular report using Ion Reporter™ Software 5.20.2.0 (Thermo Fisher Scientific). In particular, the copy number variations (CNVs) parameter related to copy gains can be strongly influenced by the tumor cellularity estimation, according to the following formula employed by the software for CNVs variant calling:

$$f*x+(1-f)*2=C \Rightarrow x=(C-(1-f)*2)/f$$

where $x$ is the value of CNVs, $f$ is the tumor cellularity (ranging from 0 to 1), and $C$ the CN observed in the sample autosome. The confidence criteria for CNVs calls employed in routine practice require the fulfillment of at least five criteria among the following: tumor cellularity ≥ 50%, minimum DNA read count threshold of 10,000 reads, median absolute pairwise difference (MAPD) ≤ 0.5, CNV confidence at 5% ≥ 4 copies, CNV level ≥ 5 copies, and a *p*-value ≤ 0.00001 of call significance, as per the manufacturer’s instructions.

### Statistical analysis

Continuous variables were summarized using mean and standard deviation (SD), as applicable, while qualitative variables were presented as counts and frequencies. To compare means and qualitative variables, *t*-tests, chi-square tests, and Mann–Whitney *U* tests were employed, depending on the nature of the data. Significance was set at *p*-values < 0.05. For assessing the correlation among different observers, vs the GT, and reliability of the cTCF obtained by different pathologists, the weighted Kappa coefficient (W*k*, quadratic function) along with its corresponding *p*-value were utilized to gauge the correlation of the percentage of tumor cellularity. Statistical analyses were performed, stratifying the groups based on the type of sample (resection, biopsies, and cell blocks), as well as categorizing samples deemed non-suitable for molecular analysis. Statistical computations were carried out using Python libraries, specifically leveraging pandas and scikit-learn.

## Results

### Cases

A total of 121 cases were used for the study. Of these, 79 (65%) were histological samples, divided into 35 (29%) surgical and 44 (36%) small bioptic specimens, and 42 (35%) were cell blocks. Details on the pTCF, cTCF and GT per sample category and final mutational status after NGS analysis are reported in Table 1.

**Table 1:** Average pTCF assessed by the three pathologists, overall and divided per sample type, along with the number of DNA/RNA mutant cases

	Surgical samples	Small biopsies	Cell blocks	Overall
Samples (*N*, %)	35 (29)	44 (36)	42 (35)	121
pTCF, Path #1 (mean ± sd)	56 (± 18)	60 (± 23)	61 (± 26)	59 (± 23)
pTCF, Path #2 (mean ± sd)	59 (± 13)	52 (± 18)	36 (± 21)	48 (± 20)
pTCF, Trainee (mean ± sd)	61 (± 15)	53 (± 23)	36 (± 22)	50 (± 23)
TCF, GT (mean ± sd)	32 (± 13)	33 (± 19)	38 (± 22)	34 (± 19)
cTCF (mean ± sd)	30 (± 9)	30 (± 10)	32 (± 11)	30 (± 10)
DNA variant detected (N, %)	26 (71)	30 (68)	28 (67)	84 (69)
RNA variant detected (N,%)	6 (17)	3 (7)	3 (7)	12 (10)
Variants	*KRAS 12, EGFR 7, MET 5, BRAF 1, ALK 1, ERBB2 1*	*KRAS 22, EGFR 6, ALK 3, BRAF 1*	*KRAS 17, EGFR 9, BRAF 4, MET 1, ALK 1*	*KRAS 51, EGFR22, MET 6, BRAF 6, ALK 5, ERBB2 1*

### pTCF inter-observer variability

The inter-observer variability among path #1, #2, and trainee, along with similar comparisons with the GT, is reported in Table 2. The overall W*k* ranged from 0.46 to 0.83 (avg. 0.59), with path #2 and trainee showing higher reproducibility as compared to path #1. The three scorers were significantly distant from the GT (overall and for each sample category, *p* < 0.01), and none of them was closer to the true tumor cellularity, as demonstrated by the overall W*k* (0.26, 0.28, and 0.26, for path #1, #2, and trainee, respectively). A total of 11 (9%), 19 (16%), and 23 (19%) cases were assigned < 20% pTCF by path #1, #2, and trainee, respectively, with only path #1 vs trainee showing statistically significant difference (*p* = 0.03, path #1 vs #2 *p* = 0.120 and #2 vs trainee *p* = 0.500). All the observers agreed on assigning < 100 cells in 12 (10%) samples, as per adequacy criteria for the molecular analysis.

**Table 2:** Inter-observer variability among pathologist #1, #2, and trainee, overall on the total samples and divided per type of specimens (surgical, small biopsy, and cell block), along with similar comparisons with the GT and significance of the pTCF differences. The reported *k* coefficients refer to the Weighted Kappa (quadratic function), while the *p*-values correspond to the *t*-test of the pTCF differences

	Overall	Surgical samples	Small biopsies	Cell blocks	*p*-value
Path #1 vs #2	0.48	0.57	0.71	0.37	< 0.001
Path #1 vs trainee	0.46	0.44	0.58	0.41	< 0.01
Path #2 vs trainee	0.83	0.68	0.78	0.83	0.640
W*k* Mean	0.59	0.57	0.69	0.54	-
Path #1 vs GT	0.26	0.12	0.31	0.23	< 0.01
Path #2 vs GT	0.28	0.12	0.35	0.39	< 0.01
Trainee vs GT	0.26	0.14	0.35	0.34	< 0.01
W*k* Mean	0.27	0.13	0.33	0.32	-

### Computational assessment of TCF

**Fig. 1:**
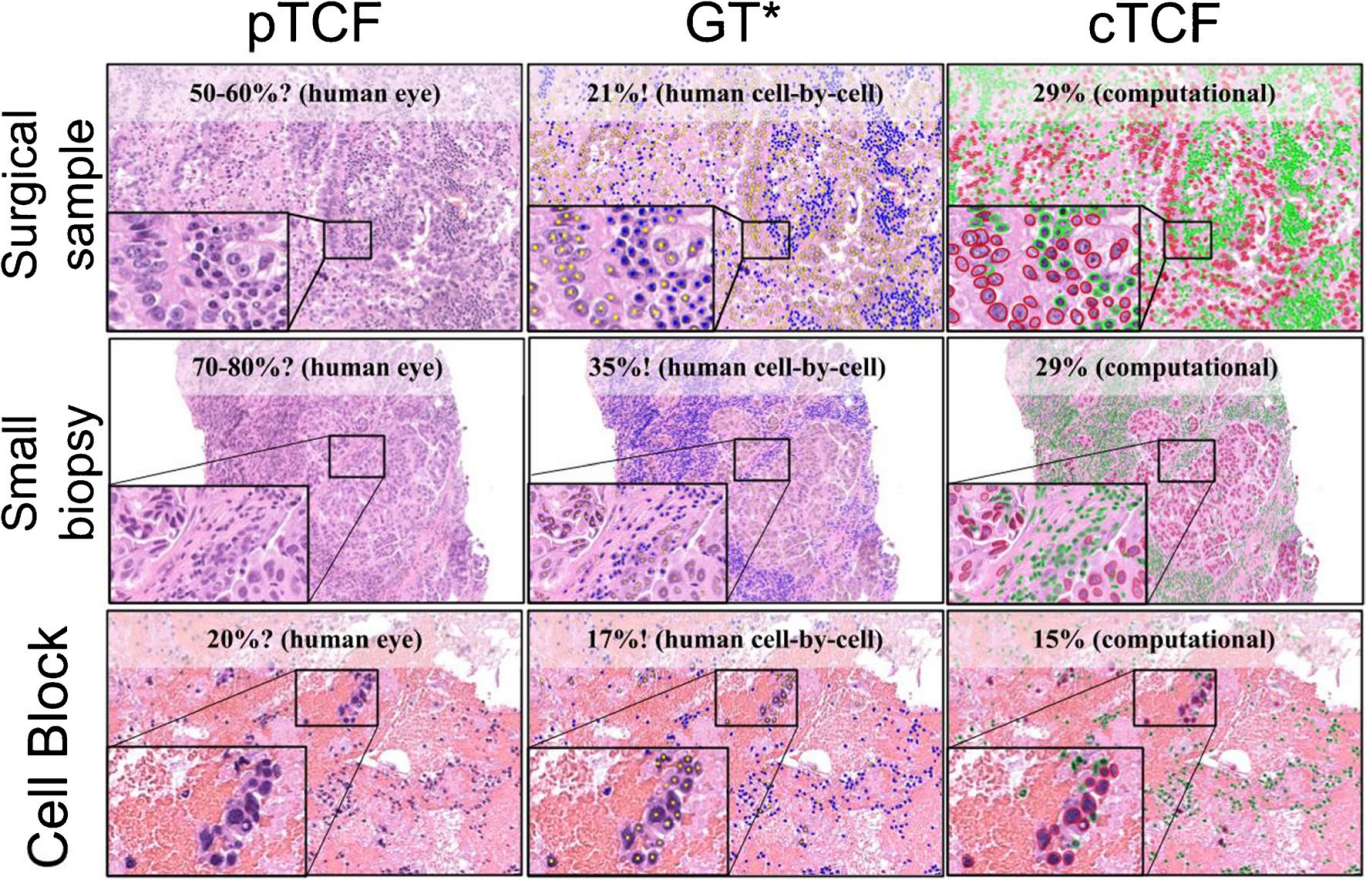
Comparative example of pTCF, GT, and cTCF on different sample types (surgical, small biopsy, and cell block). Main figure magnification × 10, inset × 40. *See Supplementary Fig. 1 for details on the GT development

The cTCF demonstrated excellent performance when compared to the GT, with no statistically significant differences (*p* = 0.129, 0.502, and 0.130 for surgical samples, small biopsies, and cell block, respectively, Fig. 1).

**Fig. 2:**
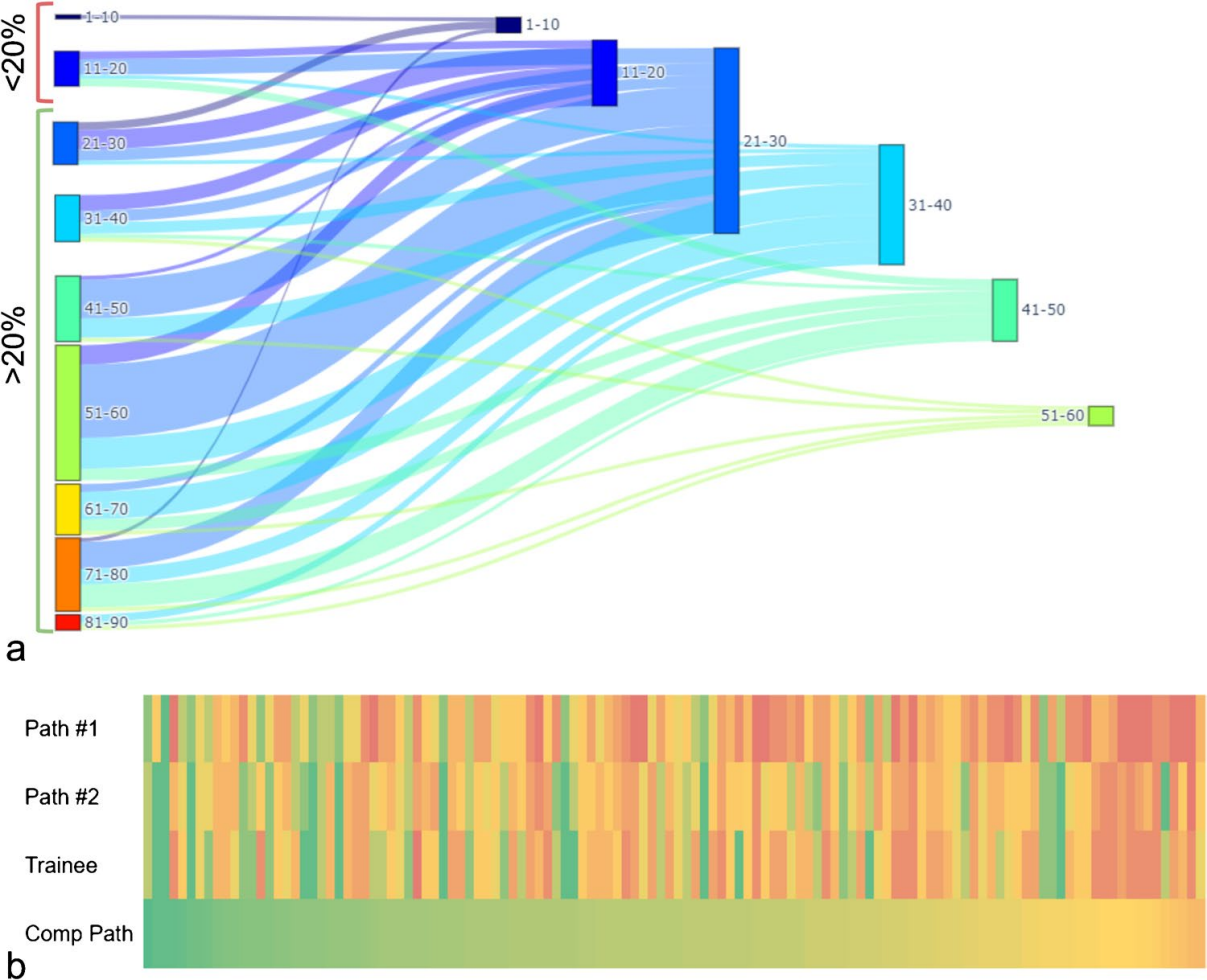
In a, cases changing TCF value from the pathologist assessment (average score, left) to the computational assessment (right). In **b,** comparative evaluation of the TCF scores given by the three pathologists and by the computational tool (green lower and red higher TCF value)

**Fig. 3:**
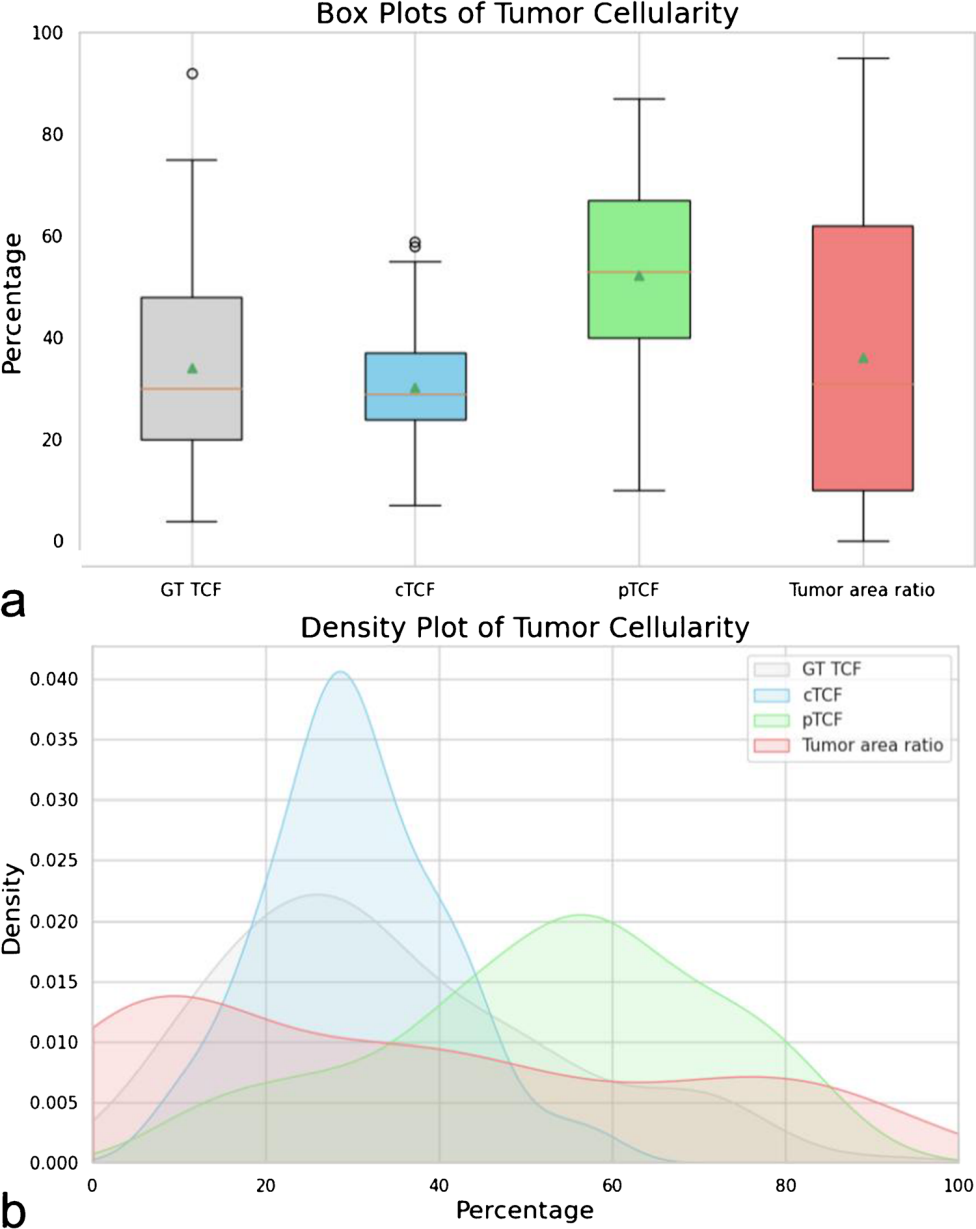
Box plot (**a**) and density plot (**b**) of tumor cellularity distribution between computational assessment, pathologists’ average score, and deep learning evaluation of tumoral area in surgical and small biopsies samples

**Fig. 4:**
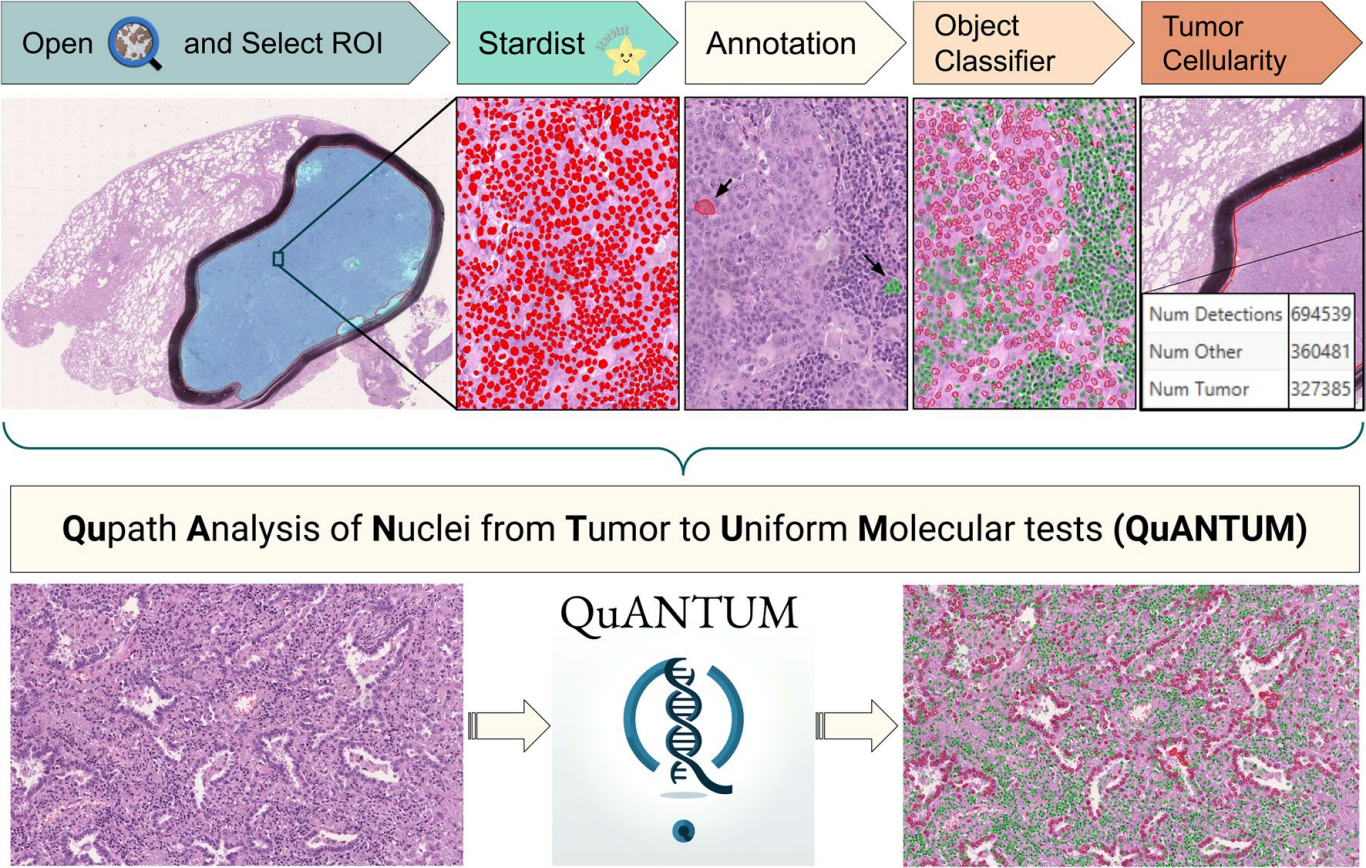
The computational pipeline used to assess TCF (QuANTUM). TCF, tumor cellular fraction; QuANTUM, Qupath Analysis of Nuclei from Tumor to Uniform Molecular tests

During the training process, an average of eight annotations (5–10) was used both for the “Tumor” and “Non-neoplastic” label, with efficacy of the model reaching performance plateau at ten annotations per category. The comparative analysis of cTCF obtained by different pathologists demonstrated substantial comparability of the results overall and for each sample type (W*k* mean = 0.9, Table 3 and Supplementary Fig. 4). After the application of the cTCF model, 106 (88%), 12 (10%), and 3 (2%) cases showed a lower, higher or equal cellularity estimation as compared to the pathologists’ average, respectively, Fig. 2. Overall, the mean cTCF was significantly lower than the pTCF (30 ± 10 vs 52 ± 19, *p* < 0.001, respectively), as confirmed by the sub-analysis on surgical, small bioptic, and cell block samples (30 ± 9 vs 59 ± 13, *p* < 0.001; 30 ± 10 vs 55 ± 19, *p* < 0.001; 32 ± 11 vs 44 ± 20, *p* = 0.001, respectively). Moreover, cTCF was < 20% in a higher number of cases, although not reaching a statistically significant difference (*p* = 0.690, Table 4). As compared to the pathologists’ assessment, none of the cases were given < 100 vital cells by the algorithm. Although these differences around the adequacy thresholds between pTCF and cTCF, a comparable rate of wild-type/mutant cases in groups with TCFs < and ≥ 20% was noted (0.42 vs 0.45, *p* = 0.810). The WSInfer DL-based assessment of tumor foci (TF) on surgical and small bioptic samples showed similarities with pTCF (*p* = 0.070) and substantial differences with the cTCF (36 ± 29, *p* < 0.001, box plots in Fig. 3a and density plots in Fig. 3b showing overlap only for pathologist assessment and tumor area). The automated cTCF pipeline have been included in a script for integration as QuPath extension (Qupath Analysis of Nuclei from Tumor to Uniform Molecular tests, QuANTUM) to streamline the process for future routine clinical applications of the computational tool (Fig. 4).

**Table 3:** Reproducibility analysis of the cTCF obtained from different pathologists, overall, and for each sample type (surgical sample, small biopsies and cell block). The reported *k* coefficients refer to the Weighted Kappa (quadratic function), while the *p*-values correspond to the Mann–Whitney *U* test of the pTCF differences

	Overall	Surgical samples	Small biopsies	Cell blocks	*p*-value
cTCF vs cTCF (CE), *k*	0.90	0.87	0.88	0.47	0.84
cTCF vs cTCF (EGR), *k*	0.88	0.64	0.84	0.67	0.87
cTCF (CE) vs (EGR), *k*	0.91	0.74	0.91	0.81	0.71
W*k* mean, *k*	0.90	0.75	0.88	0.65	-

**Table 4:** Comparison of the cases with < or ≥ 20% tumor cellularity as per cTCF, avg. pTCF, and pTCF from single pathologists, with significance of the differences. *p-values refer to the comparison of cTCF vs the other evaluations (chi-squared test)

	cTCF	pTCF avg	pTCF path #1	pTCF path #2	pTCF trainee
< 20%, *n* (%)	17 (14%)	8 (7%)	11 (9%)	19 (16%)	23 (19%)
≥ 20%, *n* (%)	104 (86%)	113 (93%)	110 (91%)	102 (84%)	98 (81%)
*p*-value*	-	0.69	0.3	0.3	0.57

### Molecular output with computational TCF

**Fig. 5:**
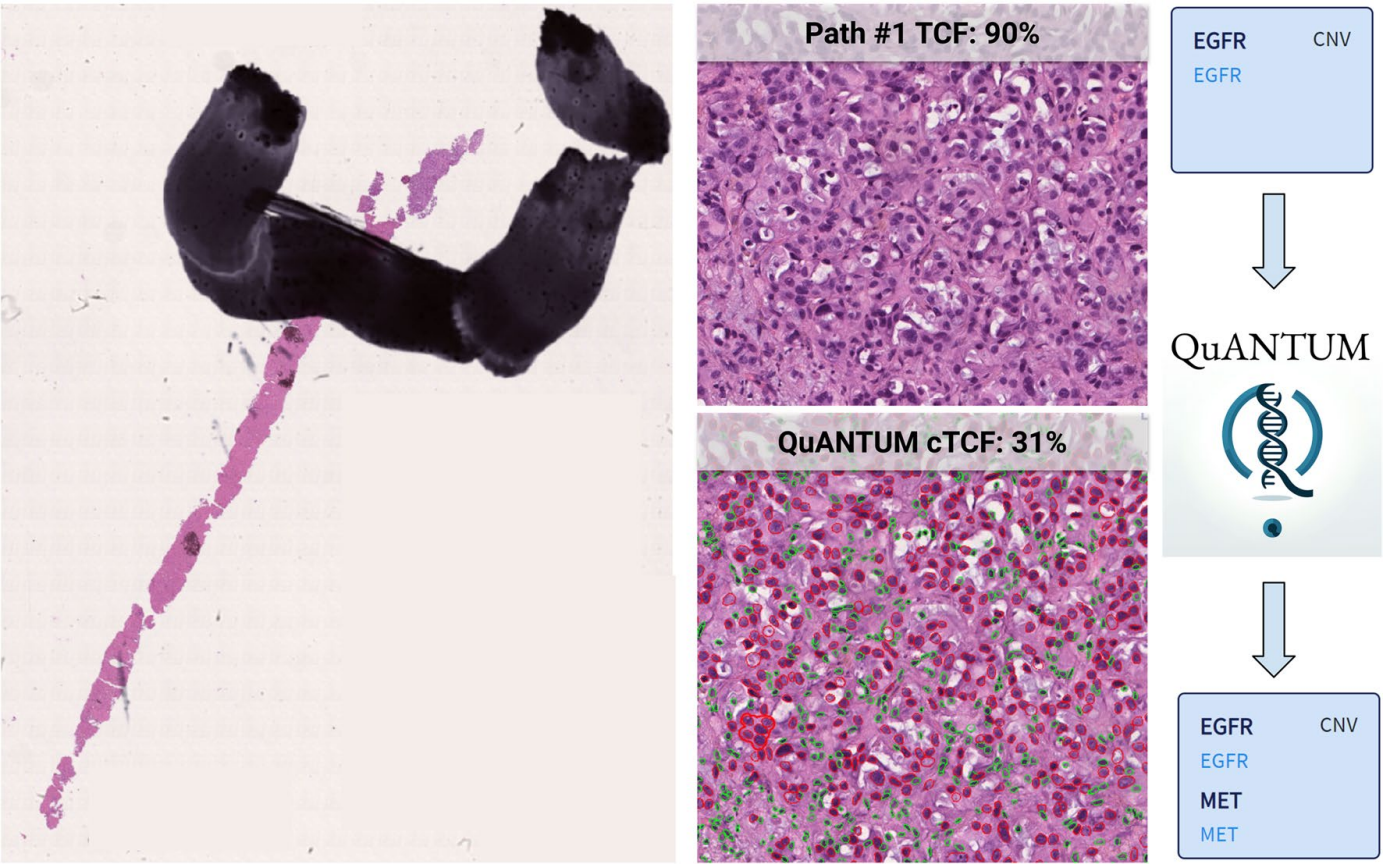
A case (n° 27 in Supplementary Table 1) undergoing a change in CNVs calling after re-assessment of TCF with QuANTUM. Based on the first evaluation by the molecular pathologist, the TCF was estimated in 90% with a consequent call of EGFR CNV. After re-evaluation based on the cTCF obtained with QuANTUM, along with the EGFR also, a MET CNV was called, with potential implications in the subsequent management of the case

A total of 29 cases (23.9%) assessed with pTCF had a CNV variant calling as per software metrics (Supplementary Table 1). From these, true CNVs were called only in seven cases with the additional confidence criteria (3 EGFR, 3 KRAS, 1 MET). After the re-evaluation with QuANTUM-derived TCF (Fig. 5), a reduction in CNVs call by the software was noted (27 vs 29, − 6.9%), with an increase in the final CNVs reported as per additional confidence criteria (13 vs 7, + 85.7%: 5 EGFR, 5 KRAS, 2 MET, 2 PIK3CA, 1 ERBB2), with 2 cases having multiple CNVs (1 case with EGFR and MET, 1 case with EGFR and PIK3CA). Thus, there was a 24% (7/29) discrepancy in final CNV calls compared to the initial analysis.

## Discussion

The recent massive introduction of predictive molecular tests in the diagnostic assessment of NSCLC stressed the need for a standardization of the preanalytical phase [28]. In this setting, one of the steps involved in the tumor tissue procurement for NGS analysis is represented by the (macro)dissection of the sample, generally performed after re-evaluation of the case by the molecular pathologist, estimating the TCF to provide the higher rate of tumor-deriving nucleic acids [29]. Previous experiences, mainly focused on the single-gene testing approach (e.g., polymerase chain reaction, PCR), led to the definition of a minimum requirement of 100 cells [18] and 20% TCF [24] as the sample adequacy criteria to obtain reliable molecular results. However, although being a relatively straightforward task for pathologists, TCF assessment still suffers from limitations mainly due to lack of clear guidelines and training sessions, heterogeneity of neoplastic cell presentation, morphology, and size and the presence of non-neoplastic cells in the tumor regions, confounding visual qualitative assessments [21, 22, 30]. These factors significantly hamper interobserver reproducibility, with up to one-third of cases being assigned ± 20% to the real TCF value in some studies [20], leading to discard about 38% following the pathologists’ TCF estimation due to the too few tumor cells for downstream molecular testing. Our study confirms this perfectible interobserver reproducibility among three different scorers (overall W*k* 0.46–0.83, avg. 0.59), with a range of “suboptimal” samples ranging from 9 to 19% (either for TCF < 20% or < 100 cells). Moreover, differences were noted among the single observer evaluations, attributable to discrepancies in training backgrounds and commitment to the TCF assessment (molecular vs general pathologist vs trainee), all three equally distant from the GT, further stressing the need for a more robust and reliable evaluation of TCF. The recent introduction of digital pathology tools is significantly twisting our practice through the progressive adoption of computer aided diagnostics (CADs), which already demonstrated their benefits in the assessment of predictive immunohistochemistry and fluorescence in situ hybridization [31, 32], pancreatic neuroendocrine tumors grading [33], and prostate cancer detection and Gleason grading [34]. In this setting, the application of CAD-based TCF assessment, associated with the experience of molecular pathologists, already demonstrated promising results in terms of reproducibility on lung adenocarcinoma [35], and the association of CAD-human assessment seems to even improve evaluation of challenging cases [23]. However, these preliminary studies were performed either on commercially available platforms that could not be widely available to general pathologists [35], or computational tools assessing cells within/outside the tumor area, potentially biased in case of highly immunogenic and lymphocyte-enriched tumors [23], with no estimation of the possible practical repercussions on the molecular results. To address the need for a democratic and user friendly QuPath-based CAD for TCF estimation, here we demonstrated reliability in the identification of tumor cells in different NSCLC sample types (surgical, small biopsies, and cell blocks), showing an overall underestimation of tumor cellularity as compared to the average pathologists’ assessment (30 ± 10 vs 52 ± 19, *p* < 0.001). This was particularly evident in surgical samples, potentially explainable by the higher influence of peri-/intratumoral background enriched in stromal and immune cells, which can act as confounding factor for the human eye and thus affecting the tumor cellularity estimation. This phenomenon has already been recognized and ascribed to the different sizes of tumor (larger) vs surrounding non-neoplastic cells, potentially leading to an overestimation of TCF by pathologists [23]. Moreover, the application of this tool showed a higher number of cases with TCF < 20% (14% vs 7% of avg. pTCF), but none with < 100 vital cells, demonstrating no significant differences in terms of mutant cases below and above the 20% threshold (0.42 vs 0.45, *p* = 0.81). These findings suggest the need for revising the currently used TCF cutoffs for material adequacy, especially if the high sensitivity and low limit-of-detection (LOD) values of the most recently introduced molecular platforms (e.g., NGS) are taken into account [36], allowing the identification of low-frequency genetic variants even on scanty cellular material. Trying to investigate the reasons leading to an overestimation of the tumor cellularity by pathologists, we explored whether the human eye can be biased by the area occupied by the tumor at low magnification, more than the actual tumor cellularity. In this direction, the application of free DL-based tools for the estimation of tumor areas (WSInfer) on surgical samples demonstrated comparability with pathologist-derived TCF (*p* = 0.07) and substantial differences with the cTCF (36 ± 29, *p* < 0.001). This is in line with the already known limitations that can affect the human interpretation of images, falling within the basket of “illusion of size” effects, consisting in the perception of an object’s size that is influenced by the context in which it is displayed [37]. This experience demonstrated a potential impact of cTCF on the CNVs calls, leading to an overall decrease in alterations detected by the variant calling software (27 vs 29, − 6.9%), but an improved detection of “true” CNVs on the final molecular report (13 vs 7, + 85.7%). In particular, while not having a strong clinical impact (CNVs are not still included in NSCLC 2023 ESCAT I-II variants), these new CNV calls could potentially candidate patients for clinical trials [38–41], or unveiling MET amplification as the cause of drug resistance in patients treated for an EGFR common mutation [42].

## Conclusion

This study confirms the reliability of computational assessment of tumor cellularity for the NGS evaluation of NSCLC specimens, applicable on both histo and cytological samples, in the setting of a second-level Molecular Pathology department. The availability of user-friendly algorithms (i.e., QuANTUM) can increase the molecular variant detection rates of our laboratories. The proposed QuANTUM computational tool can be easily used by users through the QuPath extension available at https://github.com/Gizmopath/QuANTUM.

## Supplementary Information

Below is the link to the electronic supplementary material.

Supplementary file1 (DOCX 6548 KB)

## Data Availability

The data that support the findings of this study are available from the corresponding author upon reasonable request. The QuANTUM QuPath extension is available at https://github.com/Gizmopath/QuANTUM.
