# Supplementary material for: Digital counting of tissue cells for molecular analysis: the QuANTUM pipeline
Source: Virchows Arch. 2024 Mar 26;486(2):277–86. doi: 10.1007/s00428-024-03794-9 (PMC11876257; doi:10.1007/s00428-024-03794-9)
Supplement: Supplementary file 1 — Supplementary file1 (DOCX 6548 KB) [file 428_2024_3794_MOESM1_ESM.docx]

# Supplementary Methods

*Computational pathology pipeline details*

The StarDist extension for nuclear detection has been used modifying the standard script with the following parameters: probability detection threshold of 0.2, pixel size of 0.5, and a cell constrain scale of 1.5. For the estimation of the tumor foci boundaries (TB), the deep learning (DL) WSIinfer has been applied on the same cases. In detail, the ROI corresponding to the originally annotated region was tiled using linear iterative clustering (SLIC) superpixel segmentation tool of QuPath, with the predefined set size parameters (5 μm of Gaussian sigma and 50 μm of superpixel spacing) and training parameters (number of iterations of 10 and regularization value of 0.25) before the analysis with WSInfer performed with standard lung carcinoma model (lung-tumor-resnet34.tcga-luad). Tiling was performed to make patches edge adhere to structure (e.g. neoplastic gland) boundaries as much as possible, ideally including in a tile only one type of tissue or cells, to allow WSInfer the most appropriate classification. The comparability of the obtained results among WSInfer and cTCF was obtained by merging the tumor areas detected by WSInfer under the label “Tumor” and the benign under the label “Other”. The tumor area percentage (TA, %) computed with WSInfer has been obtained by dividing the number of “Tumor” tiles by the total number of tiles into which the area was divided in the histological cohorts (n=79), excluding cell blocks for technical reasons.

*Molecular analysis*

# The automatic extraction of nucleic acids (DNA and RNA) was carried out using the Ion Torrent™ Genexus™ Purification System (GPI, Thermo Fisher Scientific, Waltham, MA, USA) with the Ion Torrent™ Genexus™ FFPE Combo Kit. Alternatively, extraction was performed using the Maxwell® CSC 16 Instrument (Promega, Madison, USA) with CSC DNA FFPE Kit or CSC RNA FFPE Kit. The GPI is equipped with a fluorimeter that automatically quantifies the extracted nucleic acid after the isolation phase, while DNA and RNA concentrations of samples extracted with the Maxwell CSC 16 instrument were determined by fluorometric quantification using the Qubit DNA dsDNA High Sensitivity Assay Kit and the Qubit RNA High Sensitivity Assay Kit on a Qubit 4.0 fluorimeter (Thermo Fisher Scientific). Analyses were performed starting with at least 10 ng of total genomic material.

# Supplementary Figure 1: Creation of the ground truth (GT) for the TCF assessment as a comparative standard to evaluate reliability of the developed computational model. For each ROI, a cell-by-cell manual classification into “Tumor”(yellow dots) and “Non neoplastic” (blue dots, e.g. immune cells, stromal cells and normal epithelial cells) categories was obtained under the supervision of two expert lung pathologists. Consequently, the TCF GT was calculated with the cell-by-cell manual classifications obtained.

#

#
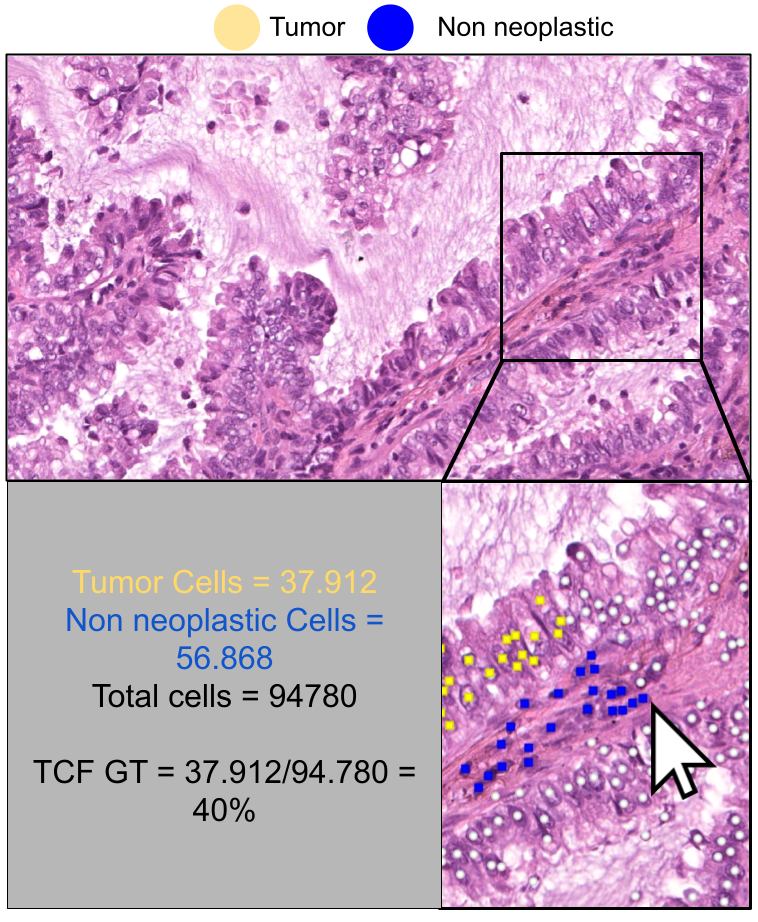


# **Supplementary Figure** 2: Comparison of the output of different machine learning algorithms used within the QuPath object classifier. In a, static pictures taken from WSI of three cases with NSCLC associated with stromal and inflammatory reaction (H&E, x20, left column). The comparison of two different ML approaches showed a substantial overestimation of tumoral cells for the KNN (right) as compared to Random Tree (center), as confirmed by the box plots (b). ML, machine learning; KNN, K-nearest neighbors.


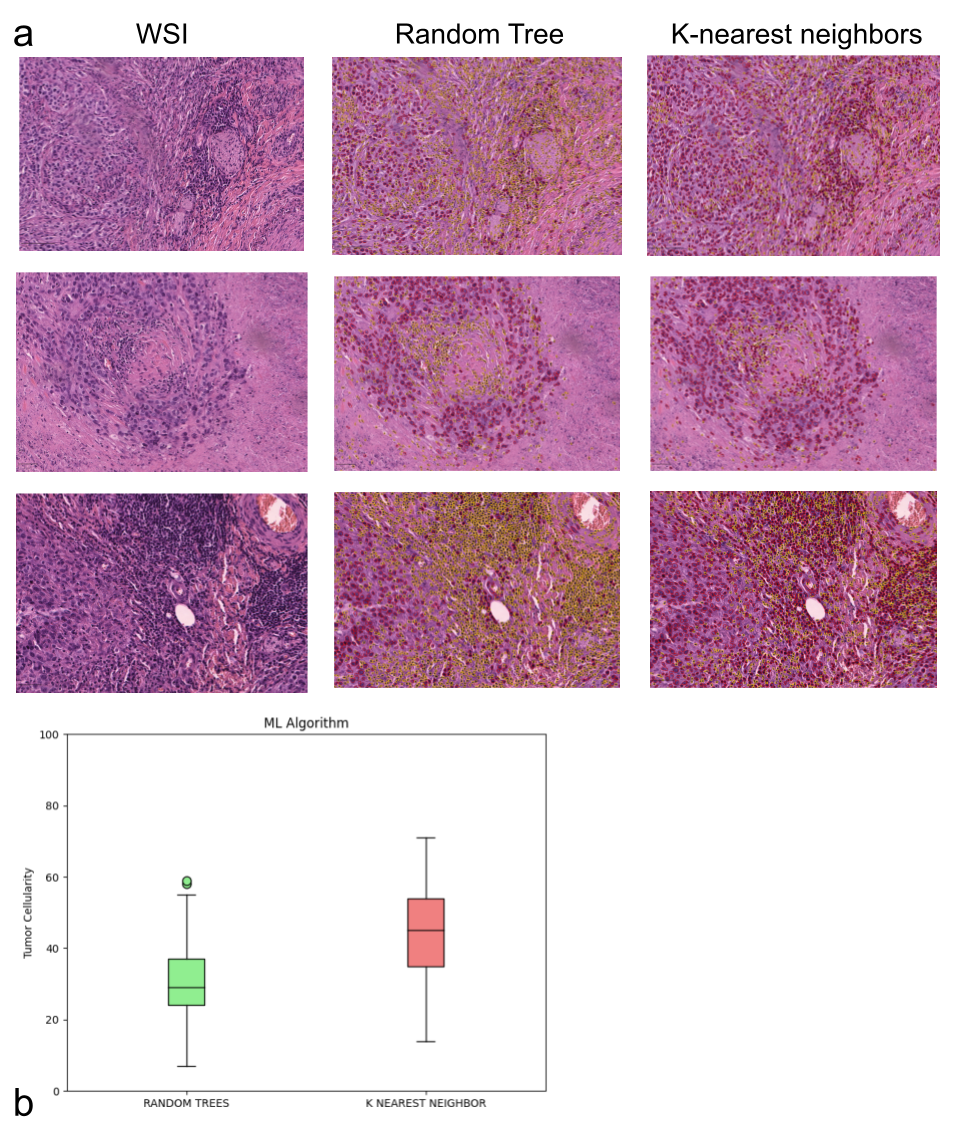


# Supplementary Figure 3: In red “Tumor” Vs “Non neoplastic” (green) nuclei. Iterative visual approach to validate the reliability of the QuPath object classifier based on RT algorithm. At progressively increasing tumor and non-tumor cells annotation, the reliability of the object classifier was increasingly high, as demonstrated by the progressively better recognition of classes by experienced pathologists visual inspection (FP and FB). cTCF, computational tumor cell fraction.


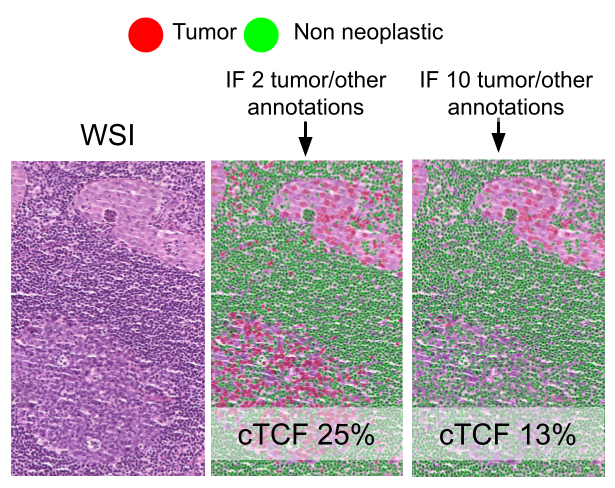


#

#

#

# Supplementary Figure 4: The comparative results obtained from the application of the cTCF pipeline by two different pathologists (validator #1 and #2) as compared to the original cTCF for the different sample types (surgical, biopsy and cell block). Main figure magnification x10, insets x40.

## **
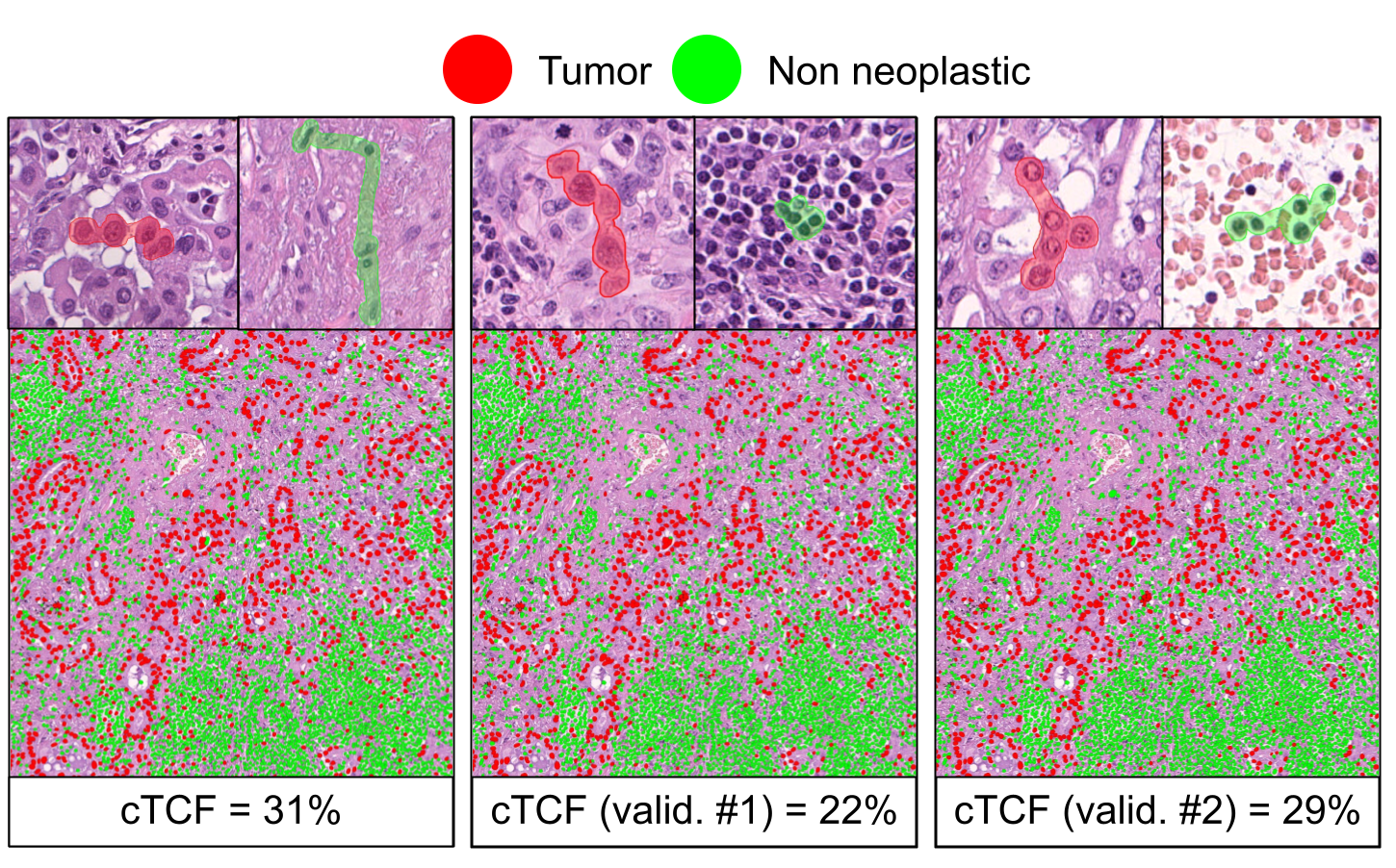
**

# Supplementary Table 1: Cases with variant calling as per software indication (n=29) with genes having CNVs evaluated based on the cellularity assessed by the pathologist #1 (yellow) and cTCF (blue). Metrics used to assess the reliability of a CNVs calling are reported, with dark red cells representing completely not fulfilled criteria, light red partially fulfilled criteria, violet cases with a change in CNVs call from path #1 to QuANTUM TCF assessment, green cases with concordant calls. The variant calls reported by the software are expressed within ESCAT I-II categories. CNV, copy number variation; MAPD, Median Absolute Pairwise Difference.

#

| Case |  | PATHOLOGIST #1 | | | | | | | | QuANTUM | | | | | | | |
| --- | --- | --- | --- | --- | --- | --- | --- | --- | --- | --- | --- | --- | --- | --- | --- | --- | --- |
|  | Software variants call | Genes with CNVs | Cellularity > 50% | DNA reads > 10.000 | MAPD< 0,5 | p-value< 0,00001 | Number of copies at 5% > 4 | Number of copies > 5 | Reported CNV (path #1 based) | Genes with CNVs | Cellularity > 50% | DNA reads > 10.000 | MAPD< 0,5 | p-value< 0,00001 | Number of copies at 5% > 4 | Number of copies > 5 | Reported CNV (QuANTUM based) |
| 1 | EGFR p.(E746_A750del) | ERBB2FGFR3KRASMETPIK3CA | 20 | YES | YES | 1,241,611,441,461,86 | 2,75,523,814,267,66 | 4,48,16,356,610,65 | NO | FGFR3KRASMETPIK3CA | 42 | YES | YES | 7,275E-60,0022363,702E-44,33E-9 | 3,682,863,084,7 | 4,94,074,196,12 | NONONOYES |
| 2 | ERBB2 p.(S310F) | FGFR3PIK3CA | 30 | YES | YES | 0,0039450,03046 | 3,062,44 | 4,774 | NO | FGFR3PIK3CACDKN2AERBB2 | 21 | YES | YES | 0,0039450,030460,040750,01353 | 3,522,622,472,82 | 5,954,864,674,71 | NO |
| 3 | / | EGFRPIK3CA | 90 | YES | YES | 2,82E-141.908E-10 | 3,713,57 | 4,384,34 | NO | EGFRPIK3CA | 55 | YES | YES | 2,82E-141,908E-10 | 4,84,57 | 5,895,84 | YESYES |
| 4 | KRAS p.(G12C) | FGFR3PIK3CA | 30 | YES | YES | 0,009550,003613 | 2,783,08 | 4,374,73 | NO | PIK3CAFGFR3PTENKRAS | 20 | YES | YES | 0,0036130,009550,075050,09288 | 3,623,172,182,03 | 6,15,554,354,45 | NO |
| 5 | KRAS p.(G12C) | PIK3CAPTEN | 20 | YES | YES | 0,01230,06181 | 2,962,25 | 5,14,25 | NO | PIK3CAFGFR3CDKN2APTENKRAS | 13 | YES | YES | 0,01230,18120,10290,061810,1651 | 3,481,4822,391,55 | 6,774,3855,464,85 | NO |
| 6 | KRAS p.(G12C) | KRAS | 90 | YES | YES | 0,00001881 | 3,25 | 4,37 | NO | PIK3CAFGFR3METPTENKRAS | 36 | YES | YES | 0,011510,0011220,012940,029611,881E-5 | 2,793,532,752,465,13 | 4,585,534,534,177,92 | NO |
| 7 | / | PIK3CA | 50 | YES | YES | 0,00002489 | 3,25 | 4,24 | NO | PIK3CAFGFR3 | 33 | YES | YES | 2,489E-50,002094 | 3,892,97 | 5,394,3 | NO |
| 8 | KRAS p.(G12C) | KRAS | 90 | YES | YES | 5,17E-10 | 3,81 | 4,78 | NO | KRAS | 53 | YES | YES | 5,17E-10 | 5,07 | 6,72 | YES |
| 9 | EGFR p.(L858R) | EGFR | 60 | YES | YES | 0,000005462 | 3,18 | 4,02 | NO | EGFR | 36 | YES | YES | 5,46E-06 | 3,96 | 5,36 | NO |
| 10 | / | KRAS | 50 | YES | YES | 2,08E-05 | 3,74 | 4,68 | NO | KRAS | 27 | YES | YES | 2,08E-08 | 5,64 | 7,58 | YES |
| 11 | KRAS p.(G12C) | FGFR3 | 30 | YES | YES | 0,00641 | 2,85 | 4,37 | NO | FGFR3 | 26 | YES | YES | 0,00641 | 2,98 | 4,73 | NO |
| 12 | / | ERBB3 | 30 | YES | YES | 0,00003733 | 3,51 | 4,7 | NO | / | 42 | YES | YES | / | / | / | / |
| 13 | / | FGFR3 | 50 | YES | YES | 0,00008488 | 3,32 | 4,54 | NO | PIK3CAFGFR3CD274PTENKRAS | 10 | YES | YES | 0,096138,488E-50,33330,21290,1852 | 2,018,60,391,041,13 | 6,614,74,65,46,1 | NO |
| 14 | KRAS p.(G12C) | PIK3CA | 20 | YES | YES | 0,02299 | 2,68 | 4,65 | NO | PIK3CA | 21 | YES | YES | 0,02299 | 2,64 | 4,52 | NO |
| 15 | EML4-ALK.E13A20.COSF408.2 | FGFR3KRAS | 10 | YES | YES | 0,1978 | 1,39 | 4,2 | NO | FGFR3KRAS | 10 | YES | YES | 0,19780,2611 | 1,391,08 | 4,24,2 | NO |
| 16 | / | FGFR3 | 20 | YES | YES | 0,009804 | 2,78 | 4,3 | NO | FGFR3 | 18 | YES | YES | 0,009804 | 2,87 | 4,56 | NO |
| 17 | / | KRAS | 30 | YES | YES | 0,00006719 | 3,76 | 5,27 | NO | KRAS | 35 | YES | YES | 6,72E-05 | 3,51 | 4,8 | NO |
| 18 | / | ERBB2 | 70 | YES | YES | 0 | 3,87 | 4,43 | NO | ERBB2 | 30 | YES | YES | 0 | 6,37 | 7,67 | YES |
| 19 | KRAS p.(G12C) | KRAS | 70 | YES | NO | 0,00309 | 2,83 | 4,23 | NO | KRAS | 26 | YES | YES | 0,00309 | 4,24 | 8 | NO |
| 20 | / | EGFR | 40 | YES | YES | 6,01E-12 | 3,58 | 4,18 | NO | EGFR | 21 | YES | YES | 6,01E-12 | 5,01 | 6,14 | YES |
| 21 | KRAS p.(G12C) | FGFR3 | 20 | YES | YES | 0,143 | 1,75 | 4,45 | NO | / | 27 | YES | YES | / | / | / | / |
| 22 | EML4-ALK.E6A20 | EGFR | 40 | YES | YES | 0,00005475 | 3,58 | 4,95 | NO | EGFR | 26 | YES | YES | 5,48E-05 | 4,43 | 6,54 | NO |
| 23 | KRAS p.(G12C) | KRAS | 90 | YES | YES | 0 | 4,13 | 4,79 | YES | FGFR3KRAS | 46 | YES | YES | 2,9E-90 | 3,696,17 | 4,57,46 | NOYES |
| 24 | / | KRAS | 70 | YES | YES | 0 | 9,57 | 11,51 | YES | FGFR3KRAS | 42 | YES | YES | 2,109E-50 | 3,3114,61 | 4,3117,86 | NOYES |
| 25 | / | EGFR | 70 | YES | YES | 0 | 8,94 | 10,04 | YES | EGFR | 24 | YES | YES | 0 | 22,24 | 25,46 | YES |
| 26 | / | MET | 40 | YES | YES | 2,22E-16 | 7,27 | 9,28 | YES | MET | 20 | YES | YES | 2,22E-16 | 12,53 | 16,55 | YES |
| 27 | EGFR p.(E746_A750del) | EGFR | 90 | YES | YES | 0 | 5,71 | 6,72 | YES | FGFR3EGFRMET | 31 | YES | YES | 1,701E-503.98E-7 | 4,2712,765,12 | 6,0615,717,1 | NOYESYES |
| 28 | / | KRAS | 90 | YES | YES | 0 | 17,42 | 20,94 | YES | KRAS | 58 | YES | YES | 0 | 25,93 | 31,4 | YES |
| 29 | EGFR p.(L747_P753delinsS) | EGFR | 80 | YES | YES | 0 | 5,16 | 5,91 | YES | EGFR | 39 | YES | YES | 0 | 8,49 | 10,03 | YES |

# 
